# Detection of kinase domain mutations in BCR::ABL1 leukemia by ultra-deep sequencing of genomic DNA

**DOI:** 10.1038/s41598-022-17271-3

**Published:** 2022-07-29

**Authors:** Ricardo Sánchez, Sara Dorado, Yanira Ruíz-Heredia, Alejandro Martín-Muñoz, Juan Manuel Rosa-Rosa, Jordi Ribera, Olga García, Ana Jimenez-Ubieto, Gonzalo Carreño-Tarragona, María Linares, Laura Rufián, Alexandra Juárez, Jaime Carrillo, María José Espino, Mercedes Cáceres, Sara Expósito, Beatriz Cuevas, Raúl Vanegas, Luis Felipe Casado, Anna Torrent, Lurdes Zamora, Santiago Mercadal, Rosa Coll, Marta Cervera, Mireia Morgades, José Ángel Hernández-Rivas, Pilar Bravo, Cristina Serí, Eduardo Anguita, Eva Barragán, Claudia Sargas, Francisca Ferrer-Marín, Jorge Sánchez-Calero, Julián Sevilla, Elena Ruíz, Lucía Villalón, María del Mar Herráez, Rosalía Riaza, Elena Magro, Juan Luis Steegman, Chongwu Wang, Paula de Toledo, Valentín García-Gutiérrez, Rosa Ayala, Josep-Maria Ribera, Santiago Barrio, Joaquín Martínez-López

**Affiliations:** 1 Hematology Department, Hospital UniversitarioHospital Universitario 12 Octubre, Madrid, Spain; 2 Instituto de Investigación Hospital 12 de Octubre (i+12), Madrid, Spain; 3 Hematological Malignancies Clinical Research Unit, CNIO, Madrid, Spain; 4 Altum Sequencing Co., Madrid, Spain; 5 Computer Science and Engineering Department, Carlos III University, Madrid, Spain; 6 Hematology Department, ICO—Hospital Germans Trias i Pujol. Josep Carreras Leukemia Research Institute, Universitat Autònoma de Barcelona, Badalona, Spain; 7 Department of Biochemistry and Molecular Biology, Pharmacy School, Universidad Complutense de Madrid, Madrid, Spain; 8 Laboratory of Neurophysiology and Synaptic Plasticity, Instituto Cajal, CSIC, Madrid, Spain; 9 Hospital Universitario de Burgos, Burgos, Spain; 10 Hospital General Universitario de Ciudad Real, Ciudad Real, Spain; 11 Hospital Virgen de la Salud, Toledo, Spain; 12 Hematology Department, ICO—Hospital Duran i Reynals (Bellvitge), Barcelona, Spain; 13 Hematology Department, ICO—Hospital Dr. Josep Trueta, Girona, Spain; 14 Hematology Department, ICO—Hospital Universitari Joan XXIII, Tarragona, Spain; 15 Hospital Universitario Infanta Leonor, Madrid, Spain; 16 Hospital Universitario de Fuenlabrada, Fuenlabrada (Madrid), Spain; 17 Hospital Central de la Defensa Gómez Ulla, Madrid, Spain; 18 Hospital Clínico San Carlos, Department of Medicine, UCM, Madrid, Spain; 19 Hospital Universitario y Politécnico La Fe, Valencia, Spain; 20 Hospital Universitario Morales-Meseguer, IMIB-Arrixaca, CIBERER, UCAM, Murcia, Spain; 21 Hospital Universitario de Móstoles, Móstoles (Madrid), Spain; 22 Hospital Universitario Niño Jesús, Madrid, Spain; 23 Hospital del Tajo, Aranjuez (Madrid), Spain; 24 Hospital Universitario Fundación Alcorcón, Alcorcón (Madrid), Spain; 25 Hospital Santa Bárbara, Puertollano, Ciudad Real Spain; 26 Hospital Universitario Severo Ochoa, Leganés, Madrid, Spain; 27 Hospital Universitario Príncipe de Asturias, Alcalá de Henares, Madrid Spain; 28 Hospital Universitario La Princesa, Madrid, Spain; 29 Hosea Precision Medical Technology Co., Ltd., Weihai, Shangdong China; 30 Hospital Universitario Ramón y Cajal, Instituto de Investigación IRYCIS, Madrid, Spain; 31 Centro de Investigación Biomédica en Red Cáncer (CIBERONC), Madrid, Spain

**Keywords:** Cancer, Molecular medicine

## Abstract

The screening of the BCR::ABL1 kinase domain (KD) mutation has become a routine analysis in case of warning/failure for chronic myeloid leukemia (CML) and B-cell precursor acute lymphoblastic leukemia (ALL) Philadelphia (Ph)-positive patients. In this study, we present a novel DNA-based next-generation sequencing (NGS) methodology for KD ABL1 mutation detection and monitoring with a 1.0E−4 sensitivity. This approach was validated with a well-stablished RNA-based nested NGS method. The correlation of both techniques for the quantification of ABL1 mutations was high (Pearson r = 0.858, p < 0.001), offering DNA-DeepNGS a sensitivity of 92% and specificity of 82%. The clinical impact was studied in a cohort of 129 patients (n = 67 for CML and n = 62 for B-ALL patients). A total of 162 samples (n = 86 CML and n = 76 B-ALL) were studied. Of them, 27 out of 86 harbored mutations (6 in warning and 21 in failure) for CML, and 13 out of 76 (2 diagnostic and 11 relapse samples) did in B-ALL patients. In addition, in four cases were detected mutation despite *BCR::ABL1* < 1%. In conclusion, we were able to detect KD ABL1 mutations with a 1.0E−4 sensitivity by NGS using DNA as starting material even in patients with low levels of disease.

## Introduction

The inclusion of BCR::ABL1 tyrosine-kinase inhibitor (TKI) in the first-line treatment of chronic myeloid leukemia (CML) and positive B-cell precursor acute lymphoblastic leukemia (ALL) with *Philadelphia* (Ph) chromosome increased the life expectancy of these patients^1,2^.

BCR::ABL1 dependent TKI resistance occurs in 10–15% of patients treated with imatinib and < 10% of patients treated with a second-generation TKI (2GTKI) as a first-line treatment^3^. These mutations affect the TKIs binding on different segments of the tertiary structure, such as the phosphate-binding loop (P-loop), ATP-binding cleft or the activation loop (A-loop). The most prevalent mutation, p.T315I, implies the change of a threonine for an isoleucine in the codon 315, preventing the correct binding of the TKI to the protein and impairing imatinib and most 2GTKI activity. The negative impact in the clinical outcome of p.T315I and other P-loop mutations including p.G250E, p.Y253H and or p.E255K/V, has been widely demonstrated^4–6^.

Besides CML, the *Ph* chromosome is also present in 25% of adult ALL patients. This alteration was formerly associated with a poor prognosis due to an increased risk of bone marrow (BM) or central nervous system (CNS) relapses. The use of TKI has significantly increased the rates of hematological responses at disease-free survival (DFS) or overall survival (OS) level^7^. The appearance of resistance is a challenge for treatment optimization. The resemblance to CML patients in blast phase (BP) suggests that the emergence of resistance to TKIs is also promoted in a large proportion of patients by mutations in the kinase domain (KD) of BCR::ABL1. However, despite low-burden BCR::ABL1 mutations are described in *Ph*-positive ALL patients, the clinical impact remains unclear. In some cases, such mutations were identified at diagnosis at subclonal level but without affecting the patient’s outcome^8–10^.

Although Sanger Sequencing (SS) remains the gold standard for diagnostic mutation screening of *Ph*-positive patients, next-generation sequencing (NGS) is replacing it, mainly due to its versatility and high sensitivity. SS is a time-consuming and low-sensitivity technique that will be presumably replaced in the next few years by NGS. Ultra-deep sequencing (UDS) by NGS can be used to track the mutation status along TKI treatment due to its high sensitivity. It can be used to discern between compound mutations or the presence of different subclones in case of multiple mutation findings^11,12^. Other techniques with similar sensitivity have served to confirm mutations in BCR::ABL1 such as digital PCR (dPCR) and allele-specific oligonucleotides-PCR (ASO-PCR)^13,14^.

Taking advantage of the *BCR::ABL1* translocation, the detection of KD mutations is mainly performed in mRNA, after enrichment of the translocation by PCR. However, RNA-based tests are sensitive to RNA denaturation and require several retro-transcription and amplification steps, source of false-positive detection induced by polymerase errors. Although DNA-based approaches for KD mutation determination have the disadvantage of amplifying *ABL1* from (i) non-rearranged *ABL1* allele of tumor cells and (ii) the *ABL1* gene of non-rearranged healthy cells, they might better represent the clonal burden and dynamics of the emerging resistant clones. Genomic DNA is more stable than RNA, and gDNA-based approaches might be applied to other dyscrasias to detect acquired mutations. Polivkova et al. studied the correlation between the digital droplet PCR (ddPCR) upon gDNA-based and mRNA-based determination for most common KD mutations with 10^–3^ sensitivity^15^. We have recently presented a DNA-based NGS pipeline to use mutations as molecular biomarkers for minimal residual disease monitoring in AML (acute myeloid leukemia) with 10^–4^ sensitivity^16^. The aim of the present study was to adapt this methodology to detect acquired *ABL1* mutations using genomic DNA as starting material and compare these results with the well-stablished RNA-based test (RNA-NestedNGS)^17^, and to explore the impact of KD mutations in patients clinical outcome.

## Results

### Screening of BCR::ABL1 KD mutation by RNA-NestedNGS

**Figure 1:**
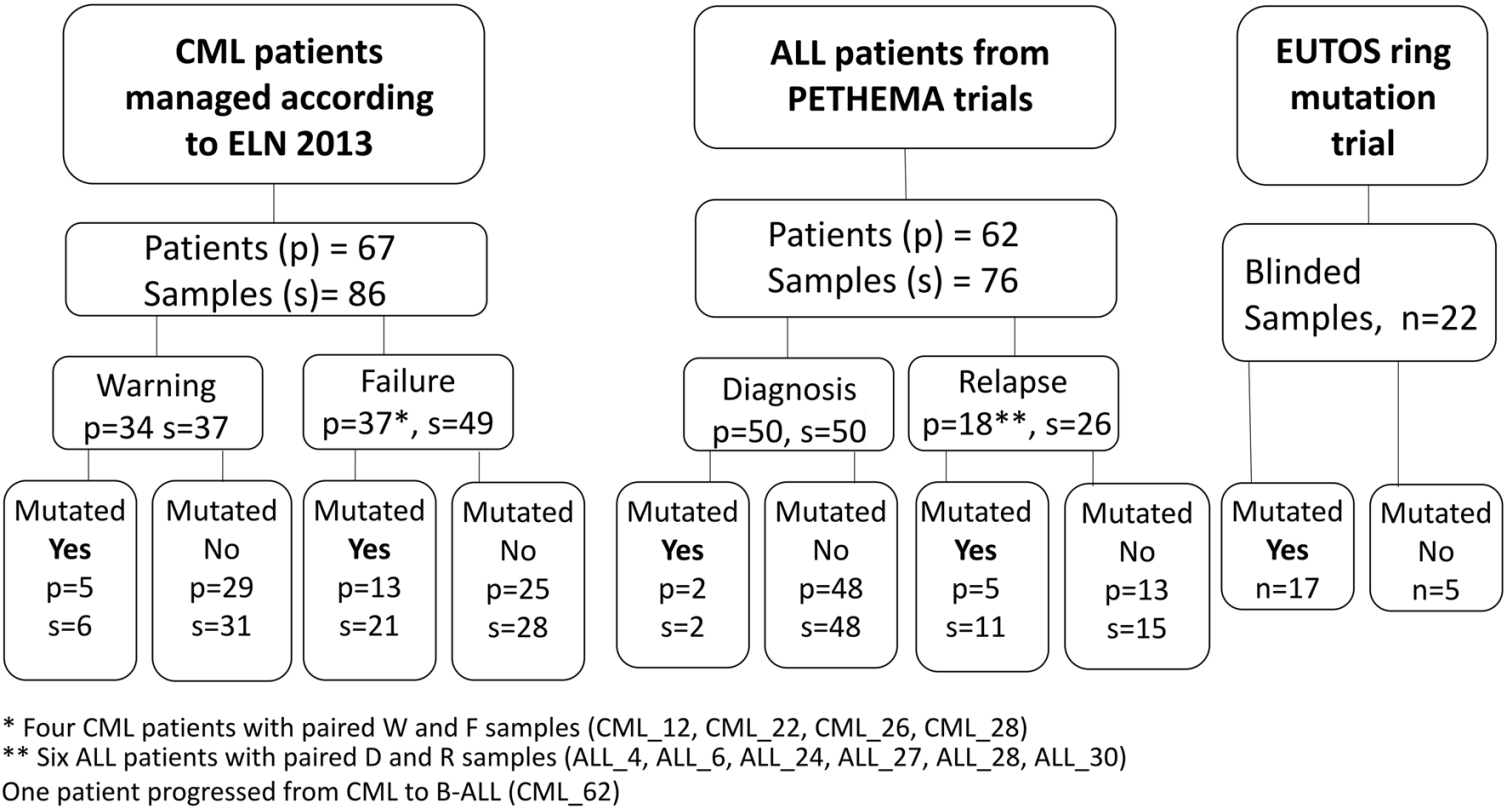
Study flow chart. Overview of the design and distribution of the patients and samples analyzed by RNA-NestedNGS. *ALL* acute lymphoblastic leukemia, *CML* chronic myeloid leukemia, *D* diagnosis, *ELN* European leukemia net, *F* failure, *R* relapse, *W* warning.

**Figure 2:**
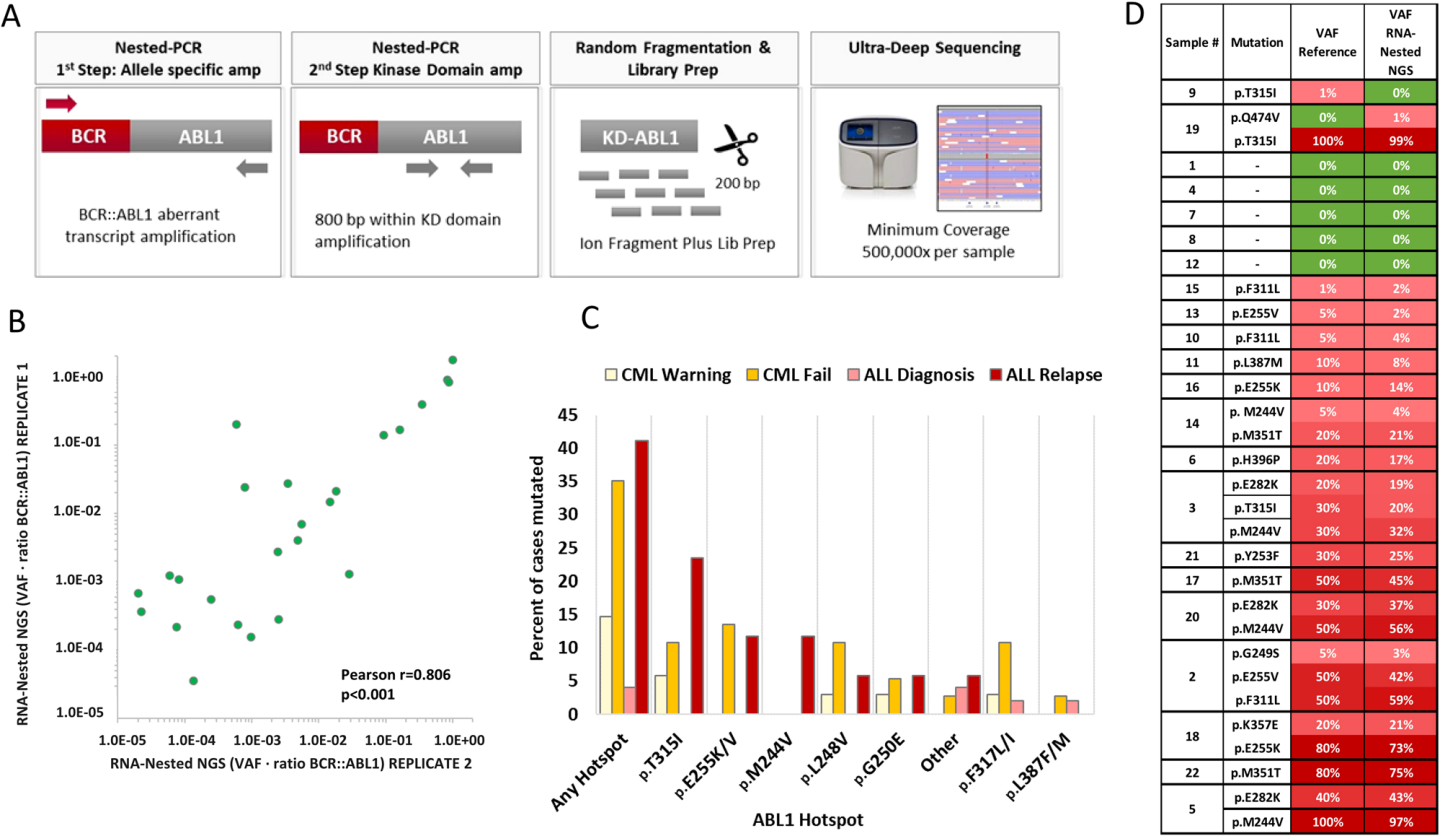
(**A**) Scheme methodology of the detection of BCR::ABL1 mutations by RNA-NestedNGS; (**B**) Correlation between the two replicates by RNA-NestedNGS; (**C**) Histogram of mutational frequency by RNA-NestedNGS method in failure/warning stages for CML patients and diagnosis/relapse ALL patients; (**D**) Results from EUTOS international control round for deep sequencing analysis of BCR::ABL1 mutations using RNA-NestedNGS approach showing the ability of the nested method to detect KD mutations. *ALL* acute lymphoblastic leukemia, *CML* chronic myeloid leukemia, *KD* kinase domain.

A total of 162 samples corresponding to 67 CML (86 samples) and 62 ALL patients (76 samples) were studied (Fig. 1). All samples were screened with an in-house RNA-NestedNGS method designed to amplify the entire KD of the rearranged allele and then sequenced by ultra-deep NGS after random enzymatic cleavage (Fig. 2A). In total, 25% samples (40/162) presented point mutations in the BCR::ABL1 KD. All samples were analyzed in duplicate, corrected VAF (variant allele frequency) calculated as the value of the VAF multiplied by the levels of *BCR::ABL1*) showed a Pearson correlation of biological duplicates for the in-house RNA-based method of 0.806 (p < 0.001, Fig. 2B). The LOD (limit of detection) of the RNA-NestedNGS is 1.0E−5 (1.0E−2 defined by the Thermo Fisher platform plus 1.0E−3 which is the minimum *BCR::ABL1* ratio level to perform the analysis). Regarding CML samples, 31% (27/86) presented KD mutations (6/37 in warning and 21/49 in failure). The remaining 13 mutated samples corresponded to ALL patients (Fig. 2C). In addition, 15 samples mutated by RNA-NestedNGS were analyzed by SS methodology. Only four were detected by SS. Eight of the mutations not detected presented a VAF below < 20%, but the remaining three were not detected despite raising VAF above the limits of SS (Supplementary Table S1).

Seven out of forty mutated samples presented at least two mutations (18%); however, compound mutations were confirmed in only one case (p.G250E and p.E255V). Notably, 6 out of 40 mutated samples corresponded to patients with low disease levels (*BCR::ABL1* < 1%).

To further investigate robustness, precision, and reproducibility of the RNA-NestedNGS approach, 22 blinded cDNA samples were provided by the EUTOS ring mutation trial for deep sequencing of BCR::ABL1 mutations. A total of 25 variants were detected in this validation cohort, including hotspots and VUS (variant of unknown significance) with VAF ranging 1–100%. Seventeen samples were mutated, and five were wild type. Only one mutation was missed by RNA-NestedNGS, affecting p.T315I (VAF = 1%). An additional false positive affecting the VUS p.Q474V (VAF of 1.13%) was called in a sample with a confirmed p.T315I (VAF = 100%, Fig. 2D).

Finally, we compared this methodology with a dPCR (digital PCR) assay for the p.T315I hotspot. To assess the performance of the dPCR assay in detecting low-frequency variants, we generated dPCR data from synthetic DNA mixtures of p.T315I mutation with VAF of 50% to 0.05%. The experimental VAFs obtained are depicted in Supplementary Fig. S1. The correlation between the dPCR measurement and the theoretical VAF, was R = 0.9999. In addition, four mutant p.T315I samples with four different VAFs covering the range found in patients were sequenced by NGS and amplified by dPCR, yielding a correlation of R = 0.9959 (Supplementary Fig. S1).

### Detection of kinase domain mutations in genomic DNA

**Figure 3:**
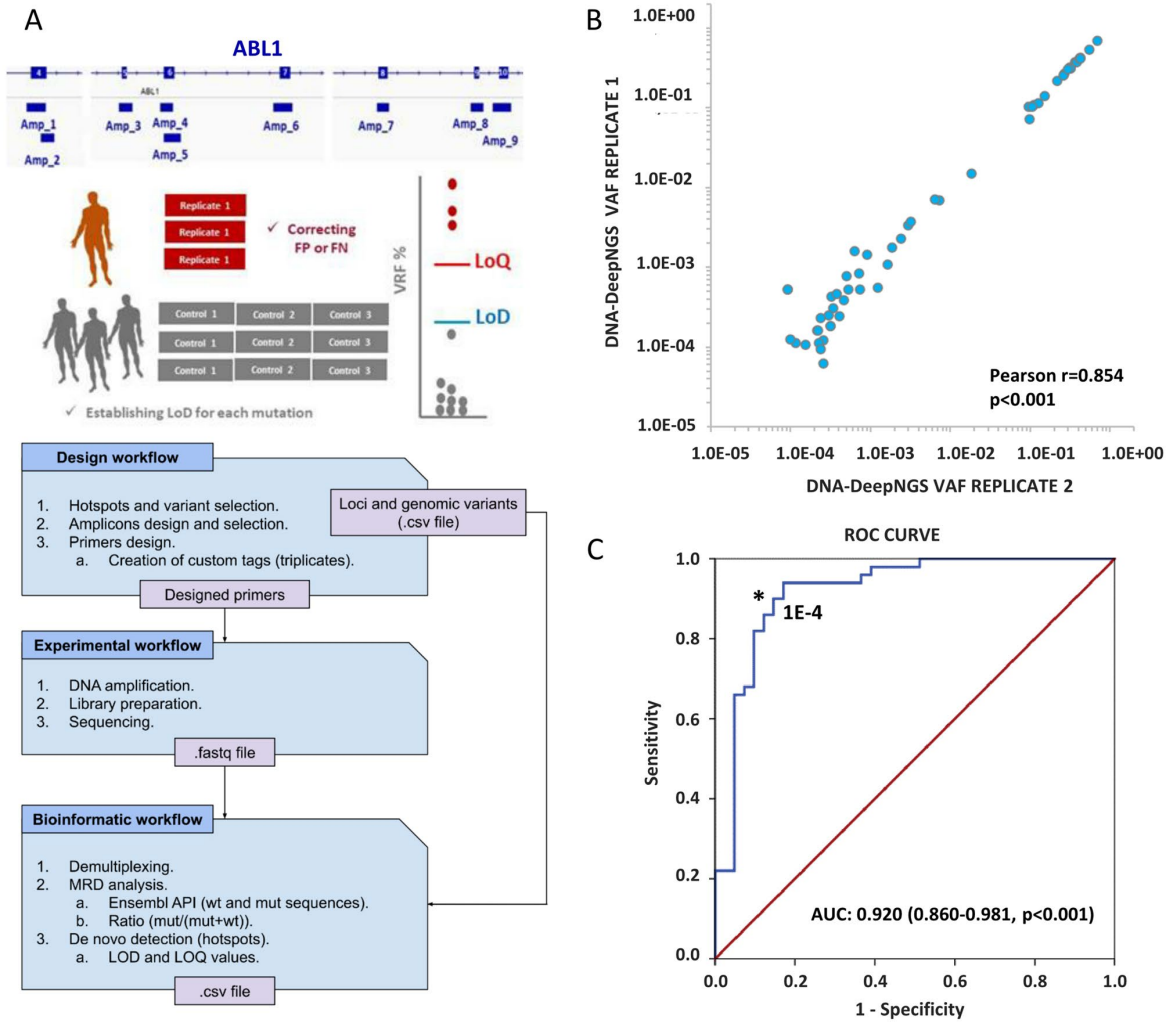
Experimental design and quality metrics resulting for DNA-DeepNGS method. (**A**) Top: nine amplicon-scheme panel designed to cover the entire KD of BCR::ABL1 and the calculation of the intrinsic error; Bottom: bioinformatic pipeline. (**B**) Correlation of two replicates for the DNA-DeepNGS approach (**C**) ROC curve comparing the two methodologies. *KD* kinase domain, *ROC* receiver operating characteristic.

To explore the viability of the gDNA detection of KD mutations, we defined an automated deep sequencing NGS-based test (DNA-DeepNGS) that includes the error correcting algorithm previously defined^16^, the sequencing of three biological replicates with a total of 500,000 × and the definition of the LOD and LOQ (limit of quantification) for every genetic position (Fig. 3A). Although this approach covers all coding regions of *ABL1* exons 4–10, we focused the study on 36 previously described hotspots (Supplementary Table S2).

First, we performed a basal noise study to calculate the LOD and LOQ of every genomic position using DNA from six healthy control donors in triplicate. The average LOD was 2.7E−5 for the 36 hotspots with only three of them (p.Y253H and p.L387H/F) with a LOD above 1.0E−4. The test was then applied to 68 samples from 46 patients previously studied by RNA-NestedNGS. The triplicates presented extraordinary reproducibility (Pearson r = 0.854, p < 0.001) for two of the triplicates (Fig. 3B) and allowed the identification and exclusion of PCR artifacts. The average curated reads obtained after applying the error corrected algorithm was 432,697 reads (rank 27,020–2,164,713). To reduce the false negative rate, a minimum of 15 mutated reads and a VAF above the LOD defined for each genetic position were required in the variant calling. With these settings the automated algorithm identified 55 potential mutations. The ROC curve of the performance for detection of KD mutations compared to the RNA-NestedNGS defined the optimum point in 1.0E−4, with an area under the curve (AUC) of 0.920 (p < 0.001, Fig. 3C). By applying this threshold, 53 mutations remained.

**Figure 4:**
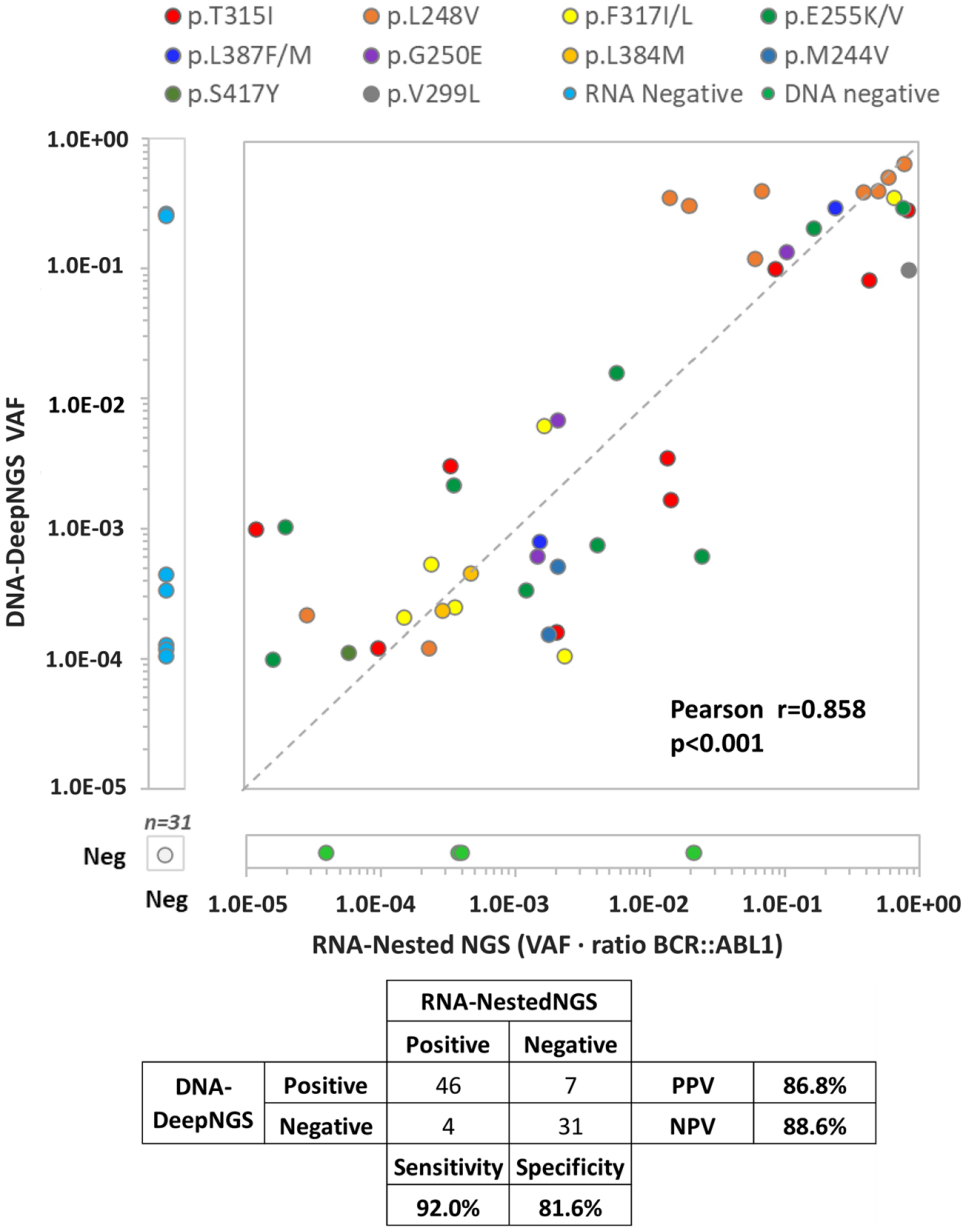
Correlation and metrics for the comparation of both methodologies. The VAF of the RNA-NestedNGS approach was corrected by the *BCR::ABL1* levels. Different hotspots are represented by different colors. The dashed line represents 100% concordance. *VAF* variant allele frequency.

The correlation of both techniques for the quantification of KD mutations was high (Pearson r = 0.858, p < 0.001) presenting a sensitivity of 92% and specificity of 81.6% for DNA-DeepNGS (Fig. 4). This comparison is possible because we corrected the tumor burden value observed in the RNA method by the disease level of each patient at that time multiplying the variant allele frequency of the mutation by the *BCR::ABL1/ABL1* ratio. Regarding the potential seven false positives of the DNA approach, two of them affected the detection of p.L248V in two samples of the same patient. This mutation was detected in six other time points by both techniques, which suggests the detection in gDNA was indeed real.

### Impact of KD mutations in the clinical outcome of the chronic myeloid leukemia patients

Clinical data of 67 CML patients in warning (n = 37) or failure (n = 34) were studied (four of them were analyzed at both time-points). Full clinical data are depicted in Supplementary Table S3A. At the time of the study 38 out of 67 CML patients were under a 2GTKI, 26 were taking imatinib and 3 ponatinib. We found mutations in 18 out of 67 patients (27%). Three of the CML patients had the unusual p190 transcript and the remaining had the p210 isoform. The median OS was 19 years. Eight out of the eighteen mutated patients carried the p.T315I mutation (44%).

**Figure 5:**
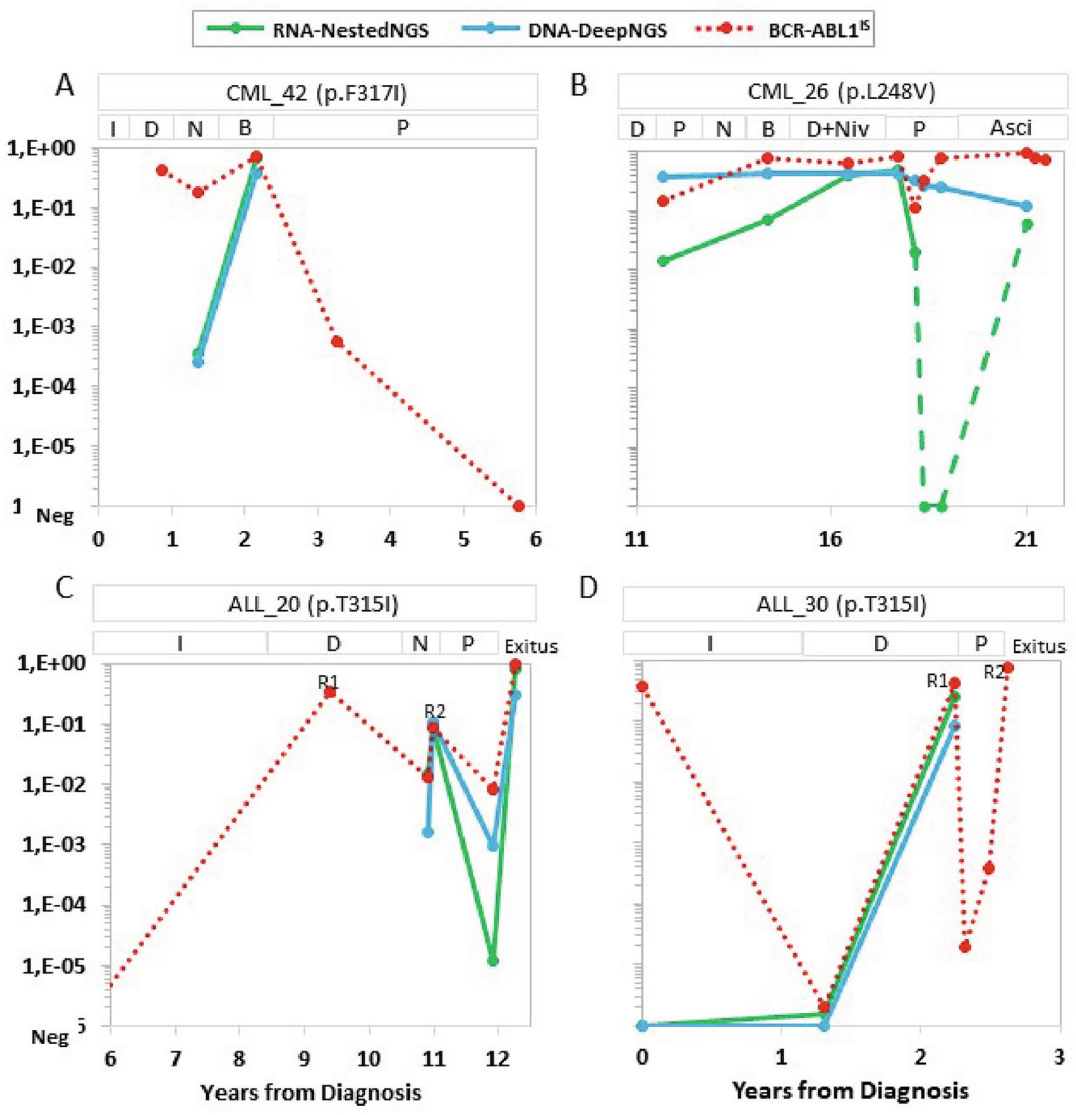
Time course for the mutations measured by DNA-DeepNGS and RNA-NestedNGS, and *BCR::ABL1* levels present in the most clinically relevant patients. Y-axis represents the value of RNA-NestedNGS VAF corrected by the ratio *BCR::ABL1* (green), DNA-DeepNGS VAF (blue) or ratio *BCR::ABL1*/*ABL1* (red). *ALL* acute lymphoblastic leukemia, *Asci* asciminib, *B* bosutinib, *CML* chronic myeloid leukemia, *D* dasatinib, *I* imatinib, *N* nilotinib, *Niv* nivolumab, *P* ponatinib, *R* relapse, *VAF* variant allele frequency.

As presented in Fig. 5, the dynamics of KD mutations in specific patients were similar in DNA and RNA. For example, the patient CML_42 acquired the p.F317I after receiving imatinib in the first line and dasatinib as the second line for 15 months. The mutation was detected in mRNA and gDNA with a corrected VAF of 4.0E−4 and 2.0E−4, respectively. After nine months under nilotinib and bosutinib, the VAF of the mutations increased three logarithms, and the *BCR::ABL1*^IS^ levels increased above 10%. Then ponatinib (15 mg/day) was started and the patient achieved grade 5 MR (molecular response) (Fig. 5A). In another patient, the clone p.L248V, described as inducing resistance to most TKIs in vitro, was detected all along the clinical history of the patient CML_26. This patient received six different TKI regimens with the *BCR::ABL1*^IS^ levels always above 1% (Fig. 5B). Although the mutation was detected by both methods, the corrected VAF in gDNA was higher than in mRNA for most time-points, and in two of them, the mutation was not detected by RNA-NestedNGS. This patient is now treated with asciminib as compassionate use, keeping in complete hematologic response (CHR).

To assess clinical impact of the mutation detection for both techniques, we defined the mutation screening as a time dependent covariate in a Cox model, confirming that CML individuals in warning or failure with KD mutations had a significantly shorter OS (HR 8.9 95% CI 2.0–39.8, p = 0.004) (Supplementary Fig. S2A).

### Impact of KD mutations in the clinical outcome of B-cell precursor acute lymphoblastic leukemia BCR::ABL1 positive

For *BCR::ABL1* positive B-ALL patients, we obtained clinical data for 62 patients, of whom 45 had p190 transcript, and 17 had p210. Clinical data are depicted in Supplementary Table S3B.

At diagnosis only 4% (2/50) of ALL patients presented mutations. This number increased to 28% in relapse (5/18). For example, the p.L387M mutation was detected in cerebrospinal fluid (CSF) blasts of patient ALL_20 in after first relapse^18^. The CSF blasts disappeared after triple intrathecal therapy and rescue chemotherapy with dasatinib. After 2 months of dasatinib treatment, therapy was suspended due to gastrointestinal toxicity. Treatment was switched to nilotinib, and a new relapse was detected one year later. Then, the p.T315I mutation was detected by both approaches, and treatment was switched again to ponatinib and stopped 18 months later by several adverse effects. The patient died 3 months later (Fig. 5C). Patient ALL_30 was diagnosed of Ph-positive ALL, with *BCR::ABL1* levels of 38% and no mutations. After 27 months with imatinib and dasatinib with undetectable *BCR::ABL1* levels, the patient relapsed and the p.T315I mutation was detected by both techniques (Fig. 5D). Treatment was switched to ponatinib, with levels of *BCR::ABL1* below 1.0E−4. Unfortunately, the patient died eight months later in disease progression. Despite the small size of the ALL sub-cohort of relapsed patients with clinical data available (n = 13), the three patients with mutations showed a trend for shorter OS (HR, hazard ratio 6.4 95% CI 0.89–46.0.8, p = 0.065, Supplementary Fig. S2B).

## Discussion

We here report the first approach for BCR::ABL1 KD mutation screening, using DNA as source material. The study demonstrates that the DNA-deepNGS method is feasible, robust and reproducible and can be easily implemented in the laboratory routines.

The identification of acquired ABL1 KD point mutations is crucial to anticipate and overcome TKI resistance. Despite SS being the gold standard for KD mutation screening, NGS and dPCR are emerging as more sensitive techniques to detect minor subclones. However, both approaches present certain limitations. The dPCR method is limited to 10–15 mutations per experiment, and mRNA-based NGS presents a high error rate induced by the retro-transcription and amplification steps.

As an alternative to RNA-based approaches, Polivkova et al.^15^ have reported the feasibility of KD mutation detection in gDNA with 0.1% sensitivity by ddPCR (droplet digital PCR). On the other hand, we have recently developed a gDNA-based approach to detect point mutations with 0.01% (or 1.0E−4) sensitivity^16^. In the present study, we improved this pipeline by including experimental triplicates in a single assay by molecular tagging. The process was entirely automated to eliminate the need for expert bioinformatic analysis. Then, we applied the DNA-DeepNGS test to two independent cohorts of CML and Ph-positive ALL patients (Fig. 1). These cohorts included CML patients in warning and failure status to TKI, and Ph-positive ALL patients at diagnosis and at relapse and allowed to explore the entire KD mutation spectrum. To validate the applicability and define the sensitivity and specificity of the DNA-based test, we compared it to an in-house RNA-NestedNGS approach used in clinical routine in the *12 de Octubre* Hospital. This test was validated by the EUTOS ring trial for BCR::ABL1 mutations where 91% of the samples (20/22) were correctly reported in both mutation and allelic frequency (Fig. 2D). Most of the mutations observed were in failure and relapse stages for CML and B-ALL patients, respectively, as previously reported^11,19^.

Our DNA-DeepNGS method was able to detect mutations with a strong reproducibility using a threshold of VAF of 1.0E−4, based on the comparison with RNA-NestedNGS in a ROC analysis (Fig. 3). This threshold improved the previous value established by other studies^19,20^, even after correcting the VAF by the *BCR::ABL1* levels, allowing the detection of mutations even with *BCR*::*ABL1* < 1%. The sensitivity of the test compared to RNA-NestedNGS was 92%, and the specificity was 81.6%. Most of false positive cases occur in samples with low *BCR::ABL1* ratios, confirming the limitations of RNA-based approaches for low tumor burden^19^ (Fig. 4). The correlation between DNA and RNA implies that resistant mutations mainly occur in the rearranged *BCR::ABL1* allele. The applicability of the DNA-DeepNGS method was 100%, being suitable for studying mutations in all samples, whereas up to 5% of the RNA samples did not present the minimum quality requirements for downstream analysis, defined by qPCR of housekeeping genes.

We found mutations in 27% of CML patients, the mutation frequency being 15% for warning and 35% for failure to TKI, which is in accordance with the results previously reported^19^. Although the detection of acquired mutations has led to a TKI change, reducing *BCR::ABL1* levels of some patients, a shorter OS of patients with mutations was observed (Supplementary Fig. 2A). That suggests that the development of resistance mutations has an intrinsic impact on prognosis. In ALL patients, determining KD mutations in cases of relapse/refractoriness (R/R) status is essential for selecting the appropriate therapeutic alternative. We also studied the impact of KD BCR::ABL1 mutations before starting the treatment. In our cohort, 2 out of 50 newly-diagnosed B-ALL patients showed mutations, in agreement with a recent study by Soverini et al.^11^, who found 3 mutated patients out of 44 patients. This frequency increased when other more sensitive methods have been used^21,22^. Some of our patients receive asciminib as compassionate use since it has been reported to be active against the most prevalent p.T315I and other double mutations and could be an exciting option for multi-resistant patients in combination with ponatinib^23^.

Our results also confirmed that most of the mutations below 20% VAF are missed by SS. Although access to NGS for some laboratories could be difficult, it is recommended to study mutations using more sensitive techniques^24^. On the other hand, the dPCR is more sensitive than the two alternatives shown here^25^. However, this technique cannot detect de novo mutations and is limited to a handful of mutations. In contrast, more than 100 different KD mutations involving over 50 amino acids have been identified so far^26^.

Although the DNA-DeepNGS method does not select the rearranged allele, we demonstrate here that the use of molecular barcodes and error-corrected algorithms allows KD mutation detection with similar resolution to RNA based approaches. Furthermore, our pipeline analyzes each position automatically and systematically. It computes the mutational ratio, LOD and LOQ for every genetic position, significantly reducing the manual work and visual inspection that RNA-nested methods typically require. More importantly, this pipeline can be adapted for the molecular screening of resistance mutations in other dyscrasias for which RNA-based methods are not applicable.

In summary, our DNA-based methodology is comparable to available RNA-based methods in terms of sensitivity and specificity. It could be introduced in the routine clinical workout for CML and R/R *Ph*-positive ALL patients with response times of 48-72 h from the sample's arrival to the issuance of the report. These results endorse this molecular approach in further clinical protocols for adopting early clinical decisions leading to improve patient management.

## Methods

### Patients and study design

We examined *BCR::ABL1* mutations in 162 BM or PB samples (Fig. 1). Sixty-seven consecutive CML patients treated in 19 public hospitals all over Spain. Inclusion criteria were failure or warning response to any TKI, any therapy line, according to the 2013 ELN recommendations, and levels of *BCR::ABL1*^*IS*^ > 0.1% (International Scale). For B-ALL disease, 62 patients were included in two consecutive clinical trials of the ALL subgroup of PETHEMA, LAL-OPH-2007^27^ and LAL-PH-2008^28^. The *BCR::ABL1* levels in B-ALL patients were not corrected for the international standardization factor. The main clinical characteristics are included in Table 1. The study was conducted in accordance with the principles of the Declaration of Helsinki, and the project has been reported as favorable by the Ethics Committee of the Hospital 12 de Octubre (CEI number: 16/187). All patients provided written informed consent for the analysis of their biological specimens.

**Table 1:** Summary of the main data of the two cohorts.

Disease/variable	ALL patients (n = 62)
Age in years, median (range)	53 (19–74)
Sex, male/female/NA	20/33/9
*BCR::ABL1* transcript p210/p190	17/45
*BCR::ABL1* mutated Y/N	7/55

Disease/variable	CML patients (n = 67)
Age, median (range), y	49 (9–84)
Sex, male/female	44/23
CP/AP/BP, n	60/3/4
Time from diag, median (range), m	53 (4–294)
Sokal high/interm/low, n (%)	23 (34) /18 (27)/ 26 (39)
Warning/Failure, n	34/33
TKI: I/N/D/B/P, n	26/17/14/7/3
*BCR::ABL1* transcript p210/p190	64/3
*BCR::ABL1* mutated, Y/N	18/49
Achieve MMR, Y/N	25/42
Number of TKI 1/2/3/4/5, n	2/19/26/17/3

*AP* accelerated phase, *ALL* acute lymphoblastic leukemia, *BP* blast phase, *B* bosutinib, *CML* chronic myeloid leukemia, *CP* chronic phase, *D* dasatinib, *diag* diagnosis, *I* imatinib, *MMR* major molecular response, *NA* non-available data, *N* nilotinib, *P* ponatinib, *TKI* tyrosin-kinase inhibitor.

### Sanger Sequencing

SS screening of the BCR::ABL1 KD was carried out on an Applied Biosystems® 3130 Genetic Analyzer (Thermo Fisher, Palo Alto, CA) as follows: RNA was extracted from fresh BM/PB using a standard TRIzol^®^ reagent-based protocol. One µg of RNA was handled to obtain cDNA using the High-capacity cDNA Reverse Transcription Kit (Thermo Fisher, Palo Alto, CA). cDNA integrity was checked by qPCR with a *GUSB* Taqman probe (Hs00939627_m1) (Thermo Fisher, Palo Alto, CA), discarding cDNA with a Ct > 25 at a threshold of 0.1. The KD of the cDNA was amplified in two steps as previously described^29,30^. In summary, in the first step of amplification, we used 5′-CGCTGACCATCAATAAG-3′ (for *BCR* exon 13) and 5′-GTACTCACAGCCCCACGGA-3′ (for *ABL1* exon 2) as the forward and reverse primers, respectively, for p210 transcripts. In the case of p190 transcripts the forward primer was replaced by 5′-CTGGCCCAACGATGGCGA-3′ (for *BCR* exon 1). The KD was amplified from the first PCR product using the primers 5′-AAGCGCAACAAGCCCACTGTCTAT-3′ and 5′-CTTCGTCTGAGATACTGGATTCCTG-3′, which cover the entire KD, from residue Gly227 to residue Gly514.

### RNA-NestedNGS methodology

RNA extraction, retro-transcription and nested PCR were carried out in the same way as the SS. Following the manufacturer’s protocols, the generated amplicons obtained from the nested PCR were then purified with Agencourt AMPure XP Beads (Beckman Coulter, Inc., Brea, CA) at an initial ratio of 1.8 × and visualized using a Bioanalyzer 2100 (Agilent Technologies, Santa Clara, CA). Further enzymatic fragmentation of the KD was performed using Ion ShearPlus (Ion Torrent, Thermo Fisher, Palo Alto, CA) for 15 min at 37 °C to obtain ∼250 bp fragments (Fig. 2A). After another identical purification step with AMPure XP Beads, 25 µL of the purified fragments were used to ligate the P1 and Ion Xpress barcode adapters (Thermo Fisher, Palo Alto, CA) and perform nick repair reactions: 15 min at 25 °C plus 5 min at 72 °C. The resulting new fragments were purified with AMPure XP beads (1.2 × ratio) and selected with E-Gel® SizeSelect™ 2% agarose gels (Invitrogen™) in an E-Gel iBaseTM and Safe Imager with a 50 bp DNA ladder (Invitrogen™). Later, we reamplified 25 µL of the fragments with the Platinum PCR SuperMix High Fidelity and the Thermo Fisher Primer Mix. The PCR steps were initial denaturation of 5 min at 95 °C, followed by 6 cycles of 15 s at 95 °C, 15 s at 58 °C and 1 min of extension at 70 °C. Finally, the fragments were purified with beads (1.5 × ratio) and quantified with the Ion Library Quantitation Kit (Thermo Fisher, Palo Alto, CA). The final barcoded libraries were adjusted to a final concentration of 50 pM and pooled with other samples, assigning 500,000 reads per sample. Template preparation, enrichment and chip loading were performed using the Ion Chef™ system (Thermo Fisher, Palo Alto, CA). Sequencing was carried out on the Ion GeneStudio S5 system (Thermo Fisher, Palo Alto, CA) (see Fig. 2A). The raw data were aligned against an artificial genome prepared from *ABL1* exons with transcript NM_005157.5 using Torrent Suite Software v.5.12 (Thermo Fisher, Palo Alto, CA), and then the generated BAM files were visualized manually using the Integrative Genomics Viewer (IGV v.2.9.4, Broad Institute, Cambridge, MA). A hotspots bed file was loaded to facilitate the mutation findings with the more frequent mutations reported.

### Digital PCR

cDNA samples were analyzed using a commercially available custom TaqMan® assay on a QuantStudio® 3D Digital PCR System (Thermo Fisher, Palo Alto, CA) to confirm p.T315I mutation with TaqMan Assay probe/primer set: C_174580870_10 (Thermo Fisher Scientific, Palo Alto, CA), including FAM-labeled probe for p.T315I and VIC-labeled probe for wild-type *ABL1*. For the dPCR reaction, 6.25 μL of the second round of the nested PCR were mixed with 0.75 μL of the 20X TaqMan® assay and 7.5 μL of 2 × QuantStudio 3D Master Mix, in a 15 μL reaction volume. Then, 14.5 μL were loaded onto QuantStudio 3D Digital PCR 20 K chips. The cycling conditions were as follows: initial denaturation at 96 °C for 10 min, followed by 39 cycles at 56 °C for 2 min and 98 °C for 30 s and an elongation step of 60 °C for 2 min, always with the cover temperature at 70 °C. Finally, samples were maintained at 20 °C in the dark for at least 30 min, and the fluorescence was read twice. Results were analyzed using QuantStudio® 3D Analysis Suite™ Cloud Software. The dilution curve was generated from a 50% mutant of p.T315I reference standard from Horizon Discovery (Cambridge, UK).

### DNA-DeepNGS methodology and automated bioinformatic pipeline

PCR amplification was carried out with the Q5 High-Fidelity DNA Polymerase (New England Biolabs, Ipswich, MA, United States). Indexed libraries were prepared following the NEBNext® Fast DNA Library Prep Set for Ion Torrent™ (NEB) and were pooled and sequenced on the Ion GeneStudio S5 System using Ion S5 Sequencing Kit, with 750 flows and 400 bp fragments. Despite our workflow being designed to screen the 1,129 genetic positions of exons 4–10 covered by the 9 amplicon design, for this work we selected the 36 most prevalent mutations with known clinical outcome^31–33^. Biological triplicates to control amplification errors during PCR cycles were analyzed independently by our bioinformatic pipeline (Fig. 3A). To mitigate noise effects, the median of the three replicates was selected as the final ratio value. Design of triplicates was conducted bioinformatically with an in-house workflow designed in R and Bash (Fig. 3A, bottom). Triplicates were distinguished between them by adding custom tags at the beginning of the primers. Each of these primers generated three types of reads of the same region after PCR amplification and sequencing that we classified as P1, P2 and P3. This allowed us to compare results between replicates and to eliminate artifacts. NGS libraries included the sequencing of biological triplicates with an estimated depth of 500,000x. The generated fastq files were automatically analyzed via a customized bioinformatic pipeline, programmed in Python and R. During the process, firstly, the raw fastq file was pre-processed using the patient information and sample identifiers, run, barcode, amplicon and triplicate identifier. Thus, the file was demultiplexed into smaller fastq files that allowed the computational optimization of the algorithm, significantly reducing the amount of time needed to analyze each sample. Secondly, to compute the variant read frequency ratios of each position in the coding regions of the selected exons, the aligned wild-type and mutated sequences (with a margin of 15 bp, queried to Ensembl^34^ via its Python application programming interface, API) of each position were searched in the corresponding demultiplexed output file from the previous step. The number of occurrences of the wild-type and the mutation sequences allowed us to compute the VAF ratio (mutated reads/total reads (mutated + non-mutated)). Then, the VAF ratio was compared with the LOD and LOQ calculated for each hotspot independently in 3 triplicated samples of 6 healthy donors. LOD was computed as the mean ratio in control samples plus 3.5 times the standard deviation, and LOQ was computed as the mean ratio in control samples plus 10 times the standard deviation. These parameters were automatically added to the final output of our pipeline, and every hotspot.

### Statistical analyses

The Pearson correlation coefficient was computed to assess the linear relationship between the different variables under study. Univariable Cox proportional hazard regression models and Kaplan–Meier survival analysis were employed to test statistical associations between genetic findings and survival outcomes. The detection of resistant mutations was defined as a time dependent covariate in the Cox proportional hazard regression analysis. This test was employed to assess statistical associations between genetic findings and survival outcomes. Statistical calculations were performed using SPSS 22.0 (SPSS Inc, Chicago). P values ≤ 0.05 were considered significant.

### Supplementary Information

Supplementary Information 1.

Supplementary Information 2.

Supplementary Information 3.

Supplementary Information 4.

## Data Availability

The raw sequencing data were uploaded to NCBI with BioProject ID: PRJNA813136 and can be found at the following link: https://www.ncbi.nlm.nih.gov/bioproject/?term=813136.
