# Author Correction: Detection of kinase domain mutations in BCR::ABL1 leukemia by ultra-deep sequencing of genomic DNA

**DOI:** 10.1038/s41598-024-57570-5

**Published:** 2024-03-28

**Authors:** Ricardo Sánchez, Sara Dorado, Yanira Ruíz-Heredia, Alejandro Martín-Muñoz, Juan Manuel Rosa-Rosa, Jordi Ribera, Olga García, Ana Jimenez-Ubieto, Gonzalo Carreño-Tarragona, María Linares, Laura Rufián, Alexandra Juárez, Jaime Carrillo, María José Espino, Mercedes Cáceres, Sara Expósito, Beatriz Cuevas, Raúl Vanegas, Luis Felipe Casado, Anna Torrent, Lurdes Zamora, Santiago Mercadal, Rosa Coll, Marta Cervera, Mireia Morgades, José Ángel Hernández-Rivas, Pilar Bravo, Cristina Serí, Eduardo Anguita, Eva Barragán, Claudia Sargas, Francisca Ferrer-Marín, Jorge Sánchez-Calero, Julián Sevilla, Elena Ruíz, Lucía Villalón, María del Mar Herráez, Rosalía Riaza, Elena Magro, Juan Luis Steegman, Chongwu Wang, Paula de Toledo, Valentín García-Gutiérrez, Rosa Ayala, Josep-Maria Ribera, Santiago Barrio, Joaquín Martínez-López

**Affiliations:** 1 Hematology Department, Hospital UniversitarioHospital Universitario 12 Octubre, Madrid, Spain; 2 Instituto de Investigación Hospital 12 de Octubre (i+12), Madrid, Spain; 3 Hematological Malignancies Clinical Research Unit, CNIO, Madrid, Spain; 4 Altum Sequencing Co., Madrid, Spain; 5 Computer Science and Engineering Department, Carlos III University, Madrid, Spain; 6 Hematology Department, ICO—Hospital Germans Trias i Pujol. Josep Carreras Leukemia Research Institute, Universitat Autònoma de Barcelona, Badalona, Spain; 7 Department of Biochemistry and Molecular Biology, Pharmacy School, Universidad Complutense de Madrid, Madrid, Spain; 8 Laboratory of Neurophysiology and Synaptic Plasticity, Instituto Cajal, CSIC, Madrid, Spain; 9 Hospital Universitario de Burgos, Burgos, Spain; 10 Hospital General Universitario de Ciudad Real, Ciudad Real, Spain; 11 Hospital Virgen de la Salud, Toledo, Spain; 12 Hematology Department, ICO—Hospital Duran i Reynals (Bellvitge), Barcelona, Spain; 13 Hematology Department, ICO—Hospital Dr. Josep Trueta, Girona, Spain; 14 Hematology Department, ICO—Hospital Universitari Joan XXIII, Tarragona, Spain; 15 Hospital Universitario Infanta Leonor, Madrid, Spain; 16 Hospital Universitario de Fuenlabrada, Fuenlabrada (Madrid), Spain; 17 Hospital Central de la Defensa Gómez Ulla, Madrid, Spain; 18 Hospital Clínico San Carlos, Department of Medicine, UCM, Madrid, Spain; 19 Hospital Universitario y Politécnico La Fe, Valencia, Spain; 20 Hospital Universitario Morales-Meseguer, IMIB-Arrixaca, CIBERER, UCAM, Murcia, Spain; 21 Hospital Universitario de Móstoles, Móstoles (Madrid), Spain; 22 Hospital Universitario Niño Jesús, Madrid, Spain; 23 Hospital del Tajo, Aranjuez (Madrid), Spain; 24 Hospital Universitario Fundación Alcorcón, Alcorcón (Madrid), Spain; 25 Hospital Santa Bárbara, Puertollano, Ciudad Real Spain; 26 Hospital Universitario Severo Ochoa, Leganés, Madrid, Spain; 27 Hospital Universitario Príncipe de Asturias, Alcalá de Henares, Madrid Spain; 28 Hospital Universitario La Princesa, Madrid, Spain; 29 Hosea Precision Medical Technology Co., Ltd., Weihai, Shangdong China; 30 Hospital Universitario Ramón y Cajal, Instituto de Investigación IRYCIS, Madrid, Spain; 31 Centro de Investigación Biomédica en Red Cáncer (CIBERONC), Madrid, Spain

Correction to: *Scientific Reports*, 10.1038/s41598-022-17271-3 published online 29 July 2022

The original version of this Article contained an error in Figure. 5, where the colors representing “DNA-Deep NGS” and “RNA-Nested NGS” were incorrect. The original Figure. 5 and accompanying legend appear below.

**Figure 5:**
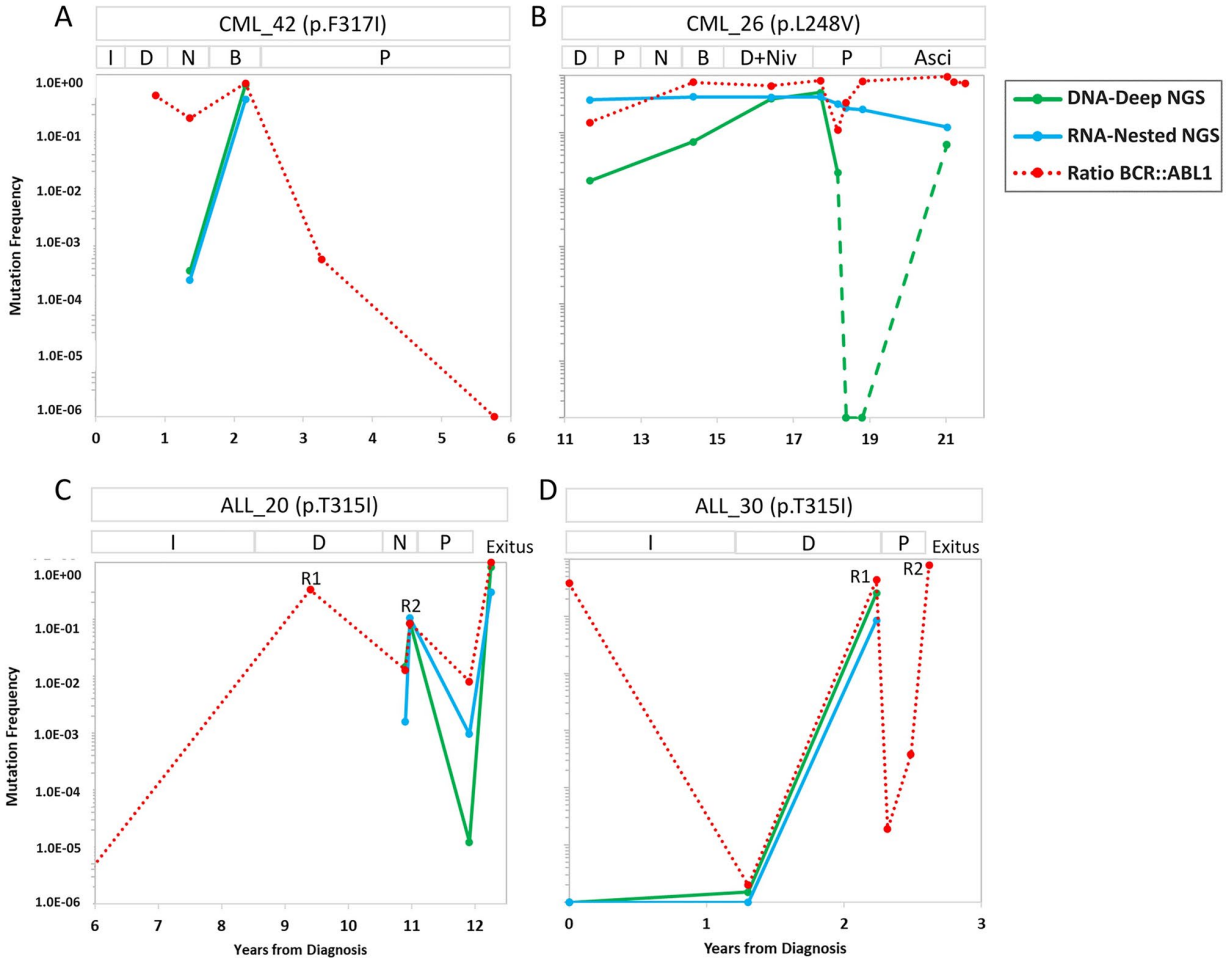
Time course for the mutations measured by DNA-DeepNGS and RNA-NestedNGS, and *BCR::ABL1* levels present in the most clinically relevant patients. Y-axis represents the value of RNA-NestedNGS VAF corrected by the ratio *BCR::ABL1* (green), DNA-DeepNGS VAF (blue) or ratio *BCR::ABL1*/*ABL1* (red). *ALL* acute lymphoblastic leukemia, *Asci* asciminib, *B* bosutinib, *CML* chronic myeloid leukemia, *D* dasatinib, *I* imatinib, *N* nilotinib, *Niv* nivolumab, *P* ponatinib, *R* relapse, *VAF* variant allele frequency.

In addition, in the Results section, under the subheading ‘Impact of KD mutations in the clinical outcome of the chronic myeloid leukemia patients’,

“The mutation was detected in mRNA and gDNA with a corrected VAF of 2.0E−4 and 4.0E−4, respectively.”

now reads:

“The mutation was detected in mRNA and gDNA with a corrected VAF of 4.0E−4 and 2.0E−4, respectively.”

The original Article has been corrected.

