# Supplementary material for: Detection of kinase domain mutations in BCR::ABL1 leukemia by ultra-deep sequencing of genomic DNA
Source: Sci Rep. 2022 Jul 29;12:13057. doi: 10.1038/s41598-022-17271-3 (PMC9338264; doi:10.1038/s41598-022-17271-3)
Supplement: Supplementary file 1 — Supplementary Information 1. [file 41598_2022_17271_MOESM1_ESM.pptx]

## Slide 1
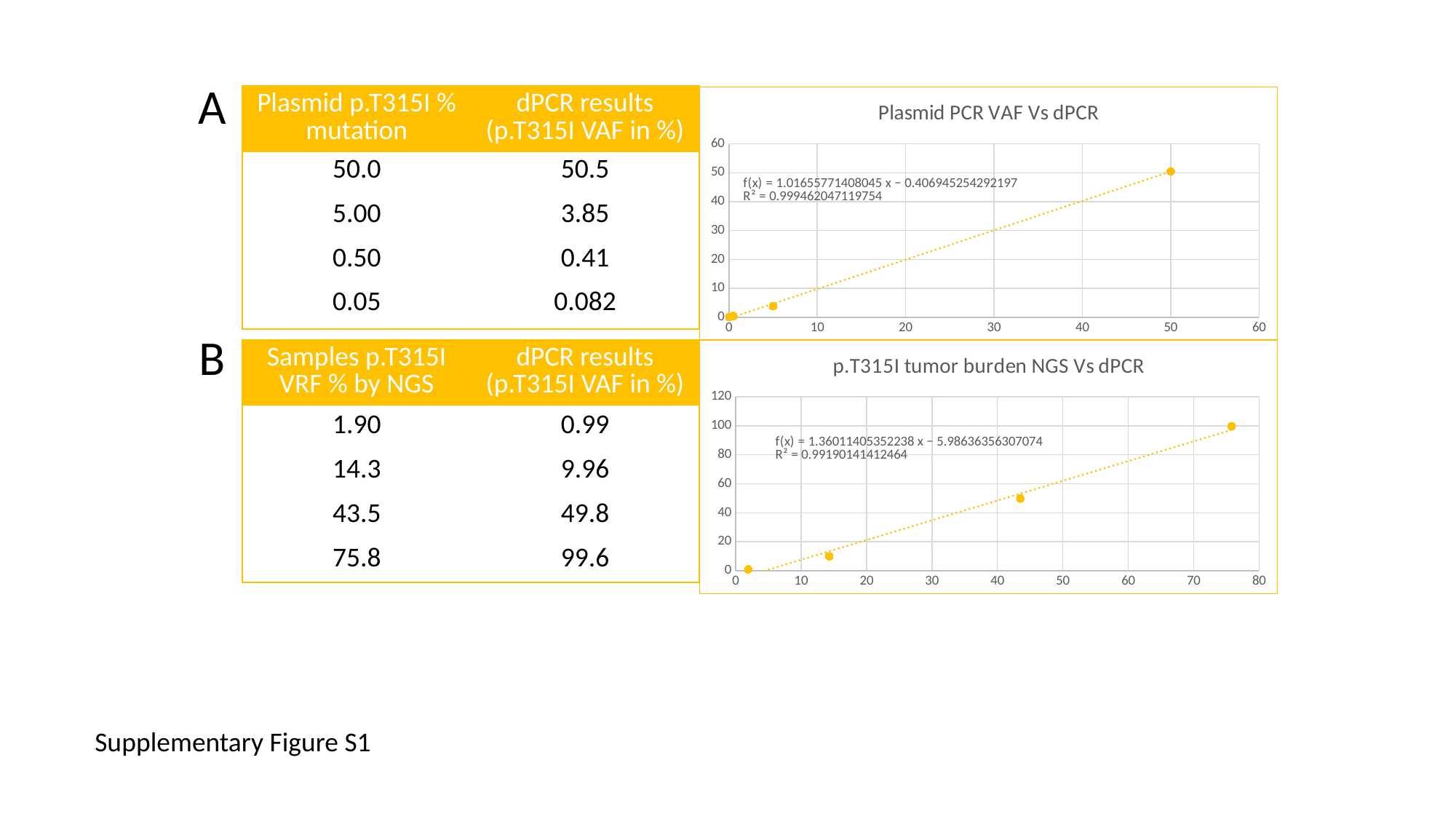

A
| Plasmid p.T315I % mutation | dPCR results (p.T315I VAF in %) |
| --- | --- |
| 50.0 | 50.5 |
| 5.00 | 3.85 |
| 0.50 | 0.41 |
| 0.05 | 0.082 |
### Chart: Plasmid PCR VAF Vs dPCR
| Category | |
|---|---|B
| Samples p.T315I VRF % by NGS | dPCR results (p.T315I VAF in %) |
| --- | --- |
| 1.90 | 0.99 |
| 14.3 | 9.96 |
| 43.5 | 49.8 |
| 75.8 | 99.6 |
### Chart: p.T315I tumor burden NGS Vs dPCR
| Category | |
|---|---|Supplementary Figure S1

## Slide 2
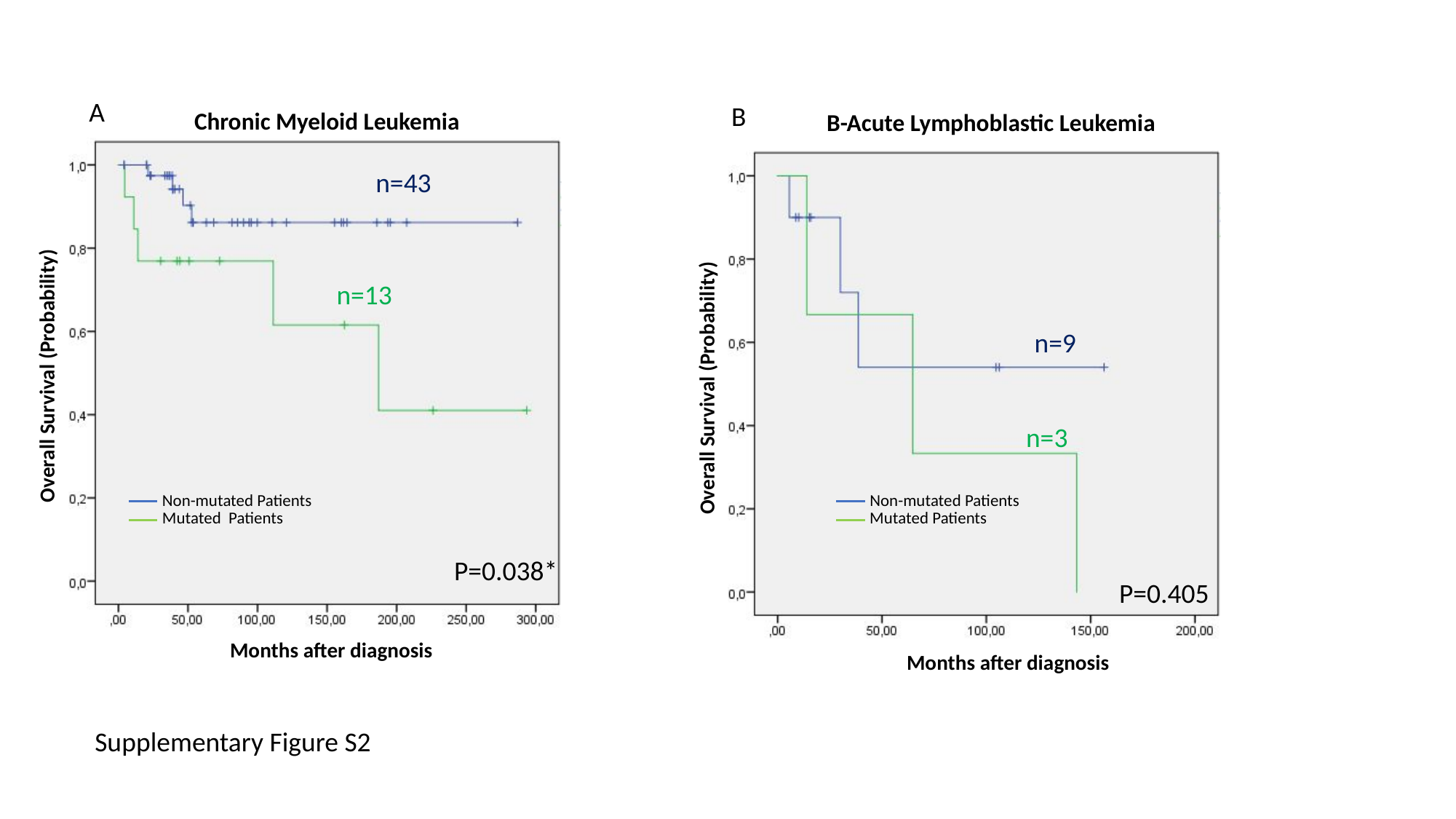

A
B
Chronic Myeloid Leukemia
B-Acute Lymphoblastic Leukemia
n=43
n=13
n=9
Overall Survival (Probability)
Overall Survival (Probability)
n=3
Non-mutated Patients
Mutated Patients
Non-mutated Patients
Mutated Patients
P=0.038*
P=0.405
Months after diagnosis
Months after diagnosis
Supplementary Figure S2
