# Supplementary material for: Detection of kinase domain mutations in BCR::ABL1 leukemia by ultra-deep sequencing of genomic DNA
Source: Sci Rep. 2022 Jul 29;12:13057. doi: 10.1038/s41598-022-17271-3 (PMC9338264; doi:10.1038/s41598-022-17271-3)
Supplement: Supplementary file 2 — Supplementary Information 2. [file 41598_2022_17271_MOESM2_ESM.pdf]

Detection of kinase domain mutations in BCR::ABL1 leukemia by ultra-deep sequencing of genomic DNA

Ricardo Sánchez, Sara Dorado, Yanira Ruíz-Heredia, Alejandro Martin, Juan Manuel Rosa-Rosa, Jordi Ribera, Olga García, Ana Jimenez-Ubieto, Gonzalo Carreño-Tarragona, María Linares, Laura Rufián, Alexandra Juárez, Jaime Carrillo, María José Espino, Mercedes Cáceres, Sara Expósito, Beatriz Cuevas, Raúl Vanegas, Luis Felipe Casado, Anna Torrent, Lurdes Zamora, Santiago Mercadal, Rosa Coll, Marta Cervera, Mireia Morgades, José Ángel Hernández-Rivas, Pilar Bravo, Cristina Serí, Eduardo Anguita, Eva Barragán, Claudia Sargas, Francisca Ferrer, Jorge Sánchez-Calero, Julián Sevilla, Elena Ruíz, Lucía Villalón, María del Mar Herráez, Rosalía Riaza, Elena Magro, Juan Luis Steegman, Chongwu Wang, Paula de Toledo, Valentín García-Gutiérrez, Rosa Ayala, Josep Maria Ribera, Santiago Barrio, Joaquín Martínez-López.

Supplementary Material

Supplementary Fig. S1. Digital PCR p.T315I validation. Correlation between (A) plasmid p.T315I 50% mutated with 3 serial dilutions vs dPCR and (B) tumor burden calculation for p.T315I mutation by NGS Vs dPCR. dPCR, digital PCR; NGS, Next-Generation Sequencing.

(As a separate Powerpoint File)

Supplementary Fig. S2. Kaplan-Meier overall survival curves for (A) CML patients, and (B) ALL patients. ALL, acute lymphoblastic leukemia; CML, chronic myeloid leukemia.

(As a separate Powerpoint File)

Supplementary Table S1. Comparative study of the mutations found by the RNA-nestedNGS method and the Sanger Sequencing method.

	Mutation RNA-NestedNGS (Yes/No)	Mutation Sanger Sequencing (Yes/No)	VAF RNA-NestedNGS
CML_6	No	No	
CML_9	Yes	No	30%
CML_18	Yes	No	40%
CML_24	Yes	No	3.0%
CML_24	Yes	No	7.0%
CML_24	Yes	Yes	29%
CML_25	No	No	
CML_25	No	No	
CML_26	Yes	No	9.5%
CML_26	Yes	No	5.0%
CML_26	Yes	No	60%

CML_27	No	No	
CML_30	Yes	No	10%
CML_39	No	No	
CML_39	No	No	
CML_45	No	No	
CML_46	Yes	Yes	47%
CML_47	No	No	
CML_51	No	No	
Control 1	No	No	
Control 2	Yes	No	3.3%
Sample 1	Yes	No	10%
Sample 2	Yes	Yes	49%
Sample 3	Yes	No	1.0%
Sample 4	No	No	
Sample 5	Yes	Yes	33%
Sample 6	No	No	
Sample 7	No	No	
Sample 8	No	No	
Sample 9	No	No	
Sample 10	No	No	

VAF variant allele frequency

Supplementary Table S2. Thirty-six mutations studied by DNA-deepNGS methodology and the reference that justifies its inclusion.

Mutation	Hotspot/Uncommon	Reference
p.M244V	Hotspot	(1)
p.L248V	Hotspot	(1)
p.L248R	Hotspot	(1)
p.G250E	Hotspot	(1)
p.Q252H	Hotspot	(1)
p.Y253H	Hotspot	(1)
p.Y253F	Hotspot	(1)
p.E255K	Hotspot	(1)
p.E255V	Hotspot	(1)
p.L273M	Uncommon mutation	(2)
p.E275K	Uncommon mutation	(3)
p.D276G	Uncommon mutation	(3)
p.T277A	Uncommon mutation	(3)
p.V299L	Hotspot	(1)
p.F311L	Hotspot	(1)
p.F311I	Uncommon mutation	(4)

p.T315I	Hotspot	(1)
p.F317V	Hotspot	(1)
p.F317I	Hotspot	(1)
p.F317L	Hotspot	(1)
p.M351T	Hotspot	(1)
p.E355G	Hotspot	(1)
p.F359V	Hotspot	(1)
p.F359C	Uncommon mutation	(3)
p.F359I	Hotspot	(1)
p.V379I	Uncommon mutation	(3)
p.L384M	Hotspot	(1)
p.L387M	Uncommon mutation	(3)
p.L387F	Uncommon mutation	(3)
p.M388L	Uncommon mutation	(3)
p.H396P	Hotspot	(1)
p.H396R	Hotspot	(1)
p.S417Y	Uncommon mutation	(3)
p.E450G	Uncommon mutation	(4)
p.E459K	Hotspot	(1)
p.F486S	Hotspot	(1)

References

1. Redaelli S, Mologni L, Rostagno R, Piazza R, Magistroni V, Ceccon M, et al. Three novel patient-derived BCR/ABL mutants show different sensitivity to second and third generation tyrosine kinase inhibitors. *Am J Hematol.* **2012**, *87*, E125-E128.

2. Jones D, Chen SS, Jabbour E, Rios MB, Kantarjian H, Cortes J. Uncommon BCR-ABL kinase domain mutations in kinase inhibitor–resistant chronic myelogenous leukemia and Ph+ acute lymphoblastic leukemia show high rates of regression, suggesting weak selective effects. *Blood*. **2010,** *115,* 5428-5429.

3. Soverini S, de Benedittis C, Mancini M, Martinelli G. Mutations in the BCR-ABL1 Kinase Domain and Elsewhere in Chronic Myeloid Leukemia. *Clin Lymphoma Myeloma Leuk*. **2015,** *15,* S120-S128.

4. Soverini S, Branford S, Nicolini FE, Talpaz M, Deininger MWN, Martinelli G, et al. Implications of BCR-ABL1 kinase domain-mediated resistance in chronic myeloid leukemia. *Leuk Res*. **2014,** *38,* 10-20.

Supplementary Table S3A. Detailed main characteristics of the CML patients.

As a separate Excel file.

Supplementary Table S3B. Detailed main characteristics of the B-ALL patients.

As a separate Excel file.
